# Transcriptional profiling of *Medicago truncatula* during *Erysiphe pisi* infection

**DOI:** 10.3389/fpls.2015.00517

**Published:** 2015-07-09

**Authors:** Miguel Curto, Franziska Krajinski, Armin Schlereth, Diego Rubiales

**Affiliations:** ^1^Department of Plant Breeding, Institute for Sustainable Agriculture, Spanish National Research CouncilCórdoba, Spain; ^2^Department of Plant-Microbe Interactions, Max Planck Institute of Molecular Plant PhysiologyPotsdam, Germany

**Keywords:** *Erysiphe pisi*, legumes, *Medicago truncatula*, transcription factors, qPCR

## Abstract

Resistance to powdery mildew has been studied in a number of plant species, yet the molecular mechanisms remain largely unknown. Transcription factors (TFs) play a critical role in the plant defense response by regulating the transcriptional machinery which coordinates the expression of a large group of genes involved in plant defense. Using high-throughput quantitative real-time PCR (qPCR) technology more than 1000 *Medicago truncatula* TFs were screened in a pair of susceptible and resistant genotypes of *M. truncatula* after 4 h of *Erysiphe pisi* infection. Seventy nine TF genes, belonging to 33 families showed a significant transcriptional change in response to *E. pisi* infection. Forty eight TF genes were differentially expressed in the resistant genotypes compared to the susceptible one in response to *E. pisi* infection, including pathogenesis-related transcriptional factors, AP2/EREBP (APETALA2/ETHYLENE-RESPONSIVE ELEMENT BINDING FACTORS), WRKY (highly conserved WRKYGQK amino-acid sequence), MYB (Myeloblastoma), homeodomain (HD) and zinc finger C_2_C_2_ (CYS_2_-CYS_2_), C_2_H_2_, (CYS_2_-HIS_2_), LIM (*L*in-11, *I*sl-1, *M*ec-3) gene families, which are involved in known defense responses. Our results suggest that these TF genes are among the *E. pisi* responsive genes in resistant *M. truncatula* that may constitute a regulatory network which controls the transcriptional changes in defense genes involved in resistance to *E. pisi*.

## Introduction

Plants grown in the natural environment are confronted by a variety of pathogens. Remaining healthy depends on their ability to recognize pathogens and to activate defense mechanisms against them. The plant defense responses are regulated by a broad number of signaling pathways. Transcription factors (TFs) control the transfer of genetic information from DNA to RNA by activation or repression of transcription, playing important roles in plant development and defense by regulating different signaling pathways (Singh et al., 2002; Udvardi et al., 2007). Data from several plant genome projects suggest that more than five percent of the plant genome encodes TF sequences (around 2000 TFs) (Riechmann and Ratcliffe, 2000). Therefore, many biologic processes, including responses to pathogens, are controlled by multiple genes managed by TFs (Singh et al., 2002). Several analyses have shown their differential expression in plants as responses to interactions with biotic and abiotic effectors (Udvardi et al., 2007). In spite of the importance of legumes as sources of protein and oil, and in the symbiotic nitrogen fixation, less than one percent of the transcript-specific regulation roles of TFs have been characterized in legumes (Udvardi et al., 2007). Among legumes, *Medicago truncatula* is a model with key attributes such as self-fertility, rapid generation time, and a small diploid genome (Singh et al., 2007) that have facilitated the use of molecular and genetic tools (Rose, 2008).

Powdery mildews are biotrophic plant pathogens that seriously constrain crop production worldwide (Bélanger et al., 2002). *Erysiphe* spp. cause considerable losses in various important legume crops (Sillero et al., 2006). This fungus has been classified into three physiologically specialized forms, f.sp. *pisi* specialized on *Pisum*, f.sp. *medicaginis* specialized on *Medicago*, and f.sp. *vicia sativa*, specialized on *Vicia* (Falloon and Viljanen-Rollinson, 2001). Breeding for powdery mildew resistance is the most desirable strategy to control this disease by means of resistant cultivars (Fondevilla and Rubiales, 2012; Rubiales et al., 2015). Consequently, several genes involved in resistance to powdery mildew have been reported in different plant species (Fondevilla et al., 2007; Yang et al., 2013; Barilli et al., 2014; Curto et al., 2015; Iglesias-García et al., 2015). High-throughput methods have resulted in identification of genes potentially associated with specific processes and characterization of the regulatory networks that control their expression (Czechowski et al., 2004; Caldana et al., 2007). Among them, DNA microarrays have been used successfully to characterize global gene expression patterns in *M. truncatula* (Foster-Hartnett et al., 2007; Samac et al., 2011; Zhang et al., 2014; Curto et al., 2015; Song et al., 2015) providing detailed information of metabolic pathways involved in the analyzed systems. Previous studies have analyzed the *E. pisi/M. truncatula* pathosystem (Foster-Hartnett et al., 2007; Samac et al., 2011; Curto et al., 2015) using different genotypes and microarray platforms, such as Mt16kOLI1, Mt16kOLI1plus (Küster et al., 2004, 2007), and Affymetrix GeneChip® (http://www.affymetrix.com). These studies have increased the knowledge of mechanisms involved in *E. pisi* resistance in *M. truncatula*, which are agreement that a wide variety of mechanisms and pathways are involved in *E. pisi* resistance including pathogenesis-related genes (i.e., *PR10, Pprg2*), as well as other genes involved in signal transduction, cell wall metabolism (i.e., *Glucan endo-1,3-beta-D-glucosidase, Pectinase*) and abiotic stress, such as *Heat shock protein 17.7*, *UVB-resistance protein BudCAR5*, and *Dehydration-responsive protein (RD22*). Although DNA microarrays have been shown to be five times less sensitive than qPCR (Czechowski et al., 2004), due to its high cost qPCR remains a technique used for low- to middle-scale studies. Several large-scale TF profiling approaches have employed the *M. truncatula* qPCR-based platform available (Kakar et al., 2008) in various studies (Verdier et al., 2008; Gao et al., 2010; Madrid et al., 2010; Villegas-Fernández et al., 2014). In this study, we screened the TF transcriptome of *M. truncatula* for altered expression during *E. pisi* infection using qPCR. Previous histological assessments showed that the resistance mechanisms carried out by the resistant genotype SA1306 is mainly related to hampering spore germination and further colony establishment by epidermal cell death as a hypersensitive response to *E. pisi* germlings that develop appressoria (Curto et al., 2015). Several mechanisms capable of monitoring changes in the plant cell wall are carried out by cellular signaling responses (Ringli, 2010; Cheung and Wu, 2011). The present study has allowed us to identify the transcription factor-encoding genes involved in the *E. pisi*/*M. truncatula* pathosystem, which are candidates for further functional studies. In addition, this approach provides a model for the regulatory network controlling the expression of TF genes in this pathosystem.

## Materials and methods

### Plant material, growth conditions, and inoculation

The study was performed through an analysis of two genotypes of *M. truncatula*, the commercial cultivar *M. truncatula* Gaertn. v. Parabinga and the accession SA1306, shown to be susceptible and resistant, respectively to *E. pisi* f.sp. *Medicaginis* (Curto et al., 2015).

The seeds of *M. truncatula* were pre-soaked in filter paper, kept in dark conditions at 4°C for 24 h, and germinated in the dark for 48 h in a growth chamber at 65% relative humidity and 20°C. The seedlings were placed in pots (125 ml) containing a 1:1 mixture of perlite and sand substrate, fertilized with half-strength Hoagland's solution (Hoagland and Arnon, 1950) 3 times a week, and grown (25°C, 12 h photoperiod, 250 μmol/m^2^ light intensity, 80% relative humidity) for 4 weeks before pathogen inoculation.

As pathogen, we used a monosporic isolate of *E. pisi* f.sp. *medicaginis* strain CO05, derived from a mildew population collected on *M. truncatula* plants at Córdoba (Prats et al., 2007), which was maintained and propagated by infecting Parabinga plants. One day before inoculation the highly infected plants were shaken to remove old conidia in order to produce an inoculum with vigorous young spores. *M. truncatula* plants of both lines were inoculated when the fourth trifoliate leaf was completely expanded (4-week-old-plant). Inoculation was carried out using a setting tower to give an inoculum density of 5 conidia mm^−2^ (Prats et al., 2007). Five plants of each *M. truncatula* genotype were inoculated per triplicate, at the same time keeping five non-infected plants as a control, in total 60 *M. truncatula* plants. Thus, three independent biological replicates, five plants per condition (control and infected) and per genotype (Parabinga and SA1306), were performed with leaflets of control and *E. pisi* infected plants of both *M. truncatula* genotypes that were harvested 4 h after *E. pisi* inoculation. The samples were immediately washed with water, blot dried with filter paper, frozen in liquid nitrogen, and stored at −80°C until RNA extraction.

### RNA extraction, cDNA synthesis, and qPCR assays

RNA was purified from collected samples using the Nucleospin RNA II kit (MACHEREY-NAGEL, Bethlehem, PA) following the manufacturer's procedure. The integrity of total RNA was assessed on 1% agarose gels (samples were denatured in formaldehyde/formamide buffer), as well as for quantity and purity by using a NanoDrop ND-100 spectrophotometer (NanoDrop Technologies, Wilmington, DE) to measure the optical density. RNA samples were digested with RNase-free DNase1 (Ambion Inc., Houston, TX), according to the manufacturer's protocol. The absence of genomic DNA was checked by PCR analysis using primers designed on the *M. truncatula* ubiquitin gene intron sequence (Kakar et al., 2008).

Synthesis of first-strand cDNA was carried out with oligo-dT12–18 (Qiagen, Hilden, Germany) using SuperScript III reverse transcriptase (Invitrogen GmbH, Karlsruhe, Germany). The efficiency of cDNA synthesis was evaluated by real-time quantitative PCR (qPCR) amplification of 5′ and 3′ regions of two reference genes, GAPDH (*Glyceraldehyde 3 phosphate dehydrogenase*) and Ubiquitin (Kakar et al., 2008). A single peak in the dissociation curve at the end of the PCR reaction allowed confirmation of the specificity of the amplified products.

A *M. truncatula* transcription factor platform composed of more than 1000 *M. truncatula* TFs gene-specific primers was used to carry out the qPCR experiments (Kakar et al., 2008). The qPCR reactions were carried out in triplicate in an optical 384-well plate with an ABI PRISM® 7900 HT Sequence Detection System (Applied Biosystems, Foster City, CA) as described previously (Kakar et al., 2008).

### Normalization and data analysis

SDS software ver. 2.3 (Applied Biosystems) was used to analyze fluorescent signals and calculate the quantification cycle (Cq) (Bustin et al., 2009). The baseline data were collected from the fluorescence signal between cycles 3 and 15, and used to correct the fluorescence signal of the samples. The PCR efficiencies (E) and correlation coefficients (*R*^2^) from linear regression analysis were calculated for each performed PCR reaction by the software LinRegPCR ver. 7.5 as described previously (Kakar et al., 2008) (Table S1). The amplification reactions with *R*^2^ < 0.99 that showed efficiencies lower than 1.8 were excluded for further analysis (24.4% of reactions). TF genes were considered detected if they were expressed in at least two biological replicates with a *Cq* < 40.

Eight reference genes encoding Pentatricopeptide repeat protein (PPRrep; TC96273), Protein phosphatase 2A subunit A3 (PDF 2; TC107161), Polypyrimidine tract-binding protein homolog (PTB; TC111751), Helicase (CB892427), Ubiquitin (TC102473), Ubiquitin-protein ligase 7 (UPL7; TC111218), Ubiquitin-conjugating enzyme E2 (UBC; AW686873), and Ubiquitin-conjugating enzyme E2 9 (UBC9; TC106312) (Kakar et al., 2008) were studied in order to determine the best suited reference genes for transcript normalization. The expression stability of the eight reference genes was analyzed by the geNorm software (Vandesompele et al., 2002; Hellemans et al., 2007) for each cDNA sample under study. In addition, pair-wise comparison analysis allowed determining the optimal number of reference genes in this assay (Vandesompele et al., 2002).

Expression values were calculated from E^Cq^ of each individual plot. To normalize the gene expression of each PCR reaction, ratios of the geometric mean of the selected reference genes to the different biological conditions were used using the Equation (1):

(1) $$\left(\frac{{\text{E}}_{{\text{ref}}^{\left(\text{Cq ref}\right)}}}{{\text{E}}_{{\text{gene}}^{\left(\text{Cq gene}\right)}}}\right)$$

The relative induction/repression of TFs from *E. pisi* infected samples compared to untreated samples was calculated using the Equation (2):

(2) $$\left(\frac{\left(\frac{{\text{E}}_{{\text{ref}}^{\left(\text{Cq ref}\right)}}}{{\text{E}}_{{\text{gene}}^{\left(\text{Cq gene}\right)}}}\right)\text{infected condition}}{\left(\frac{{\text{E}}_{{\text{ref}}^{\left(\text{Cq ref}\right)}}}{{\text{E}}_{{\text{gene}}^{\left(\text{Cq gene}\right)}}}\right)\text{untreated condition}}\right)$$

Non-parametric Levene's test and Spearman's correlation coefficient were used to verify the equality of variances in the samples and to study the similarity between TF gene expression profiles, respectively. TF genes showing statistically significant differences (*P* < 0.05) were clustered using a hierarchical cluster analysis by complete linkage. A model for the regulatory network controlling the expression of regulated genes induced by *E. pisi* in both *M. truncatula* genotypes studied was built using NodeXL (http://nodexl.codeplex.com).

## Results

### Evaluation of resistance in *Medicago truncatula* genotypes

Differences in the response to *E. pisi* between the two genotypes were not yet visible at the time the leaves were sampled for RNA extraction. Powdery mildew infection was macroscopically visible on remaining leaflets 2 weeks after inoculation, with profuse sporulation in the susceptible Parabinga genotype and absence of symptoms in the resistant SA1306 genotype (Figure S1). Thus, previous study described that colony formation was much higher in Parabinga than in SA1306, as well as the hypersensitive response associated with epidermal cell death was negligible in Parabinga, but marked in SA1306 (Curto et al., 2015). Former studies unveiled that at early *E. pisi* infection times, such as 4 h (Curto et al., 2015) and 12 h (Samac et al., 2011), *M. truncatula* plants induce a highly number of metabolic pathways in response to *E. pisi* infection. Hence, we choose to analyze the TF transcriptome of *M. truncatula* during *E. pisi* infection at 4 h after pathogen infection.

### Selection of reference genes

**Figure 1:**
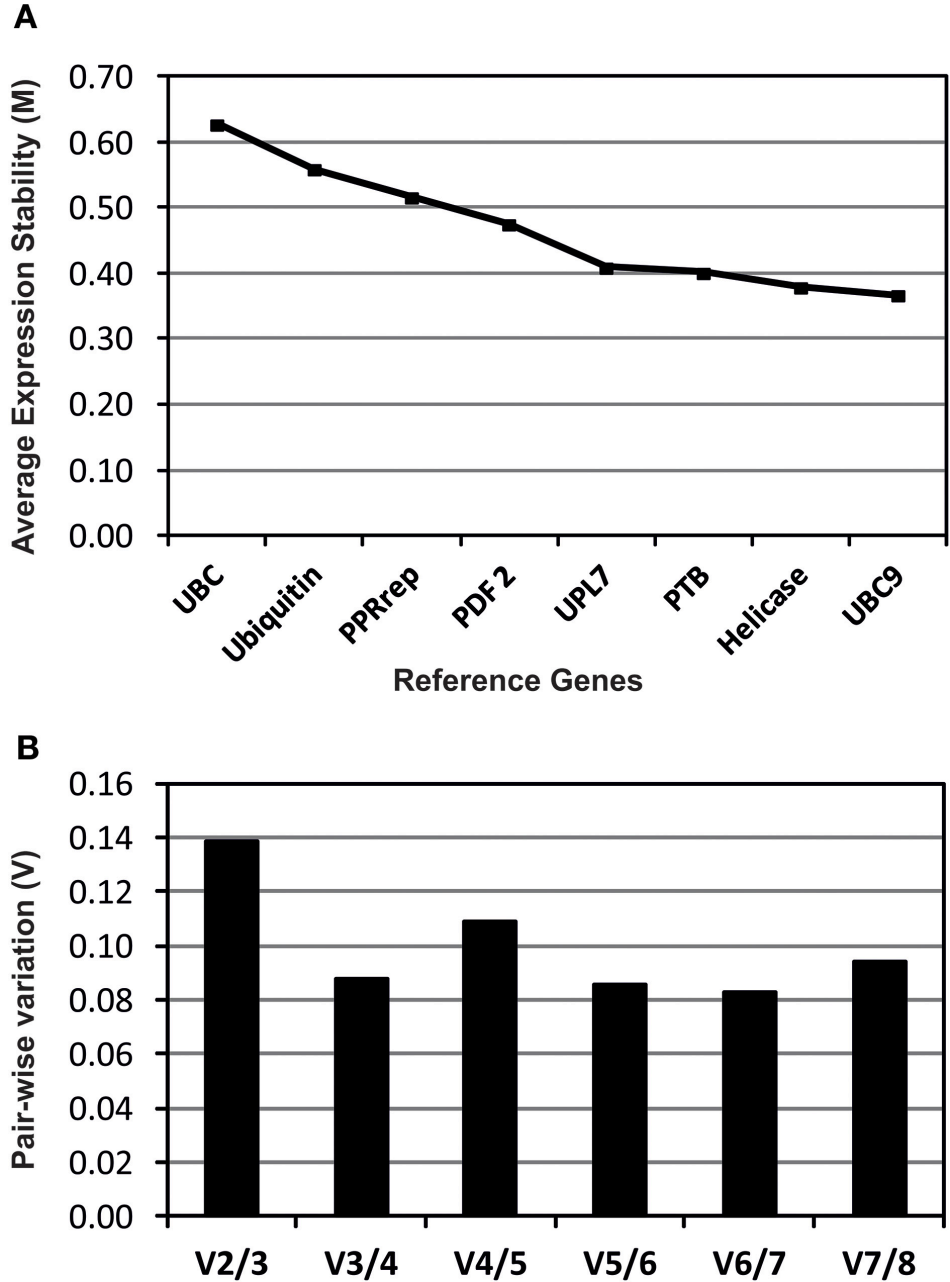
**Evaluation of candidate reference genes analyzed using geNorm software**. Expression stability **(A)** and pair-wise variation **(B)** plots for the eight reference genes studied. A lower M value indicates a more stable expression. The pair-wise variation (V) values indicate the optimal number of reference genes.

Eight reference genes were studied to determine those best suited for transcript normalization. Transcripts levels of all reference genes were calculated, in each cDNA sample, using the average expression stability (M) calculated by geNorm software (Figure 1A). All reference genes showed high average expression stability (*M* < 0.66) among them the *UBC9*, *Helicase*, *PTB*, and *UPL7* reference genes showed the lower average expression stability (M) indicating a greater transcript stability (Figure 1A). Pair-wise variation (V) was also calculated as described by Vandesompele et al. (2002) allowing determining the optimal number of stable reference genes. The results indicated that the inclusion of a third gene (V_3/4_) or more genes (V_4/5_, V_5/6,_V_6/7_, and V_7/8_) has no significant effect (Figure 1B). Therefore, we selected *UBC9* and *Helicase* as the best reference genes for this experiment, which were used for transcript normalization of the analyzed TF genes.

### Expression patterns of TF genes in *M. truncatula* following *E. pisi* infection

**Figure 2:**
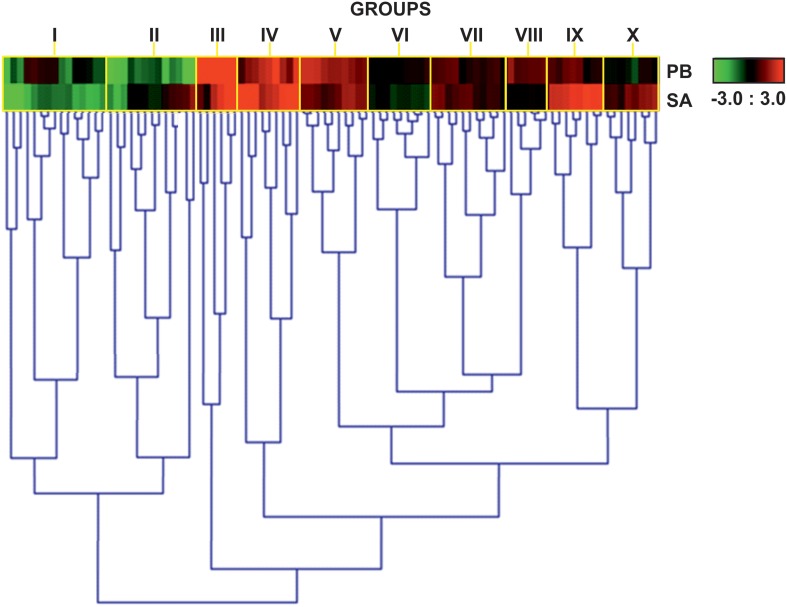
**Heat map expression profiles of TF genes**. Heat map showing expression profiles of 95 genes that were differentially expressed in Parabinga (PB) and SA1306 (SA) *M. truncatula* genotypes in response to *E. Pisi* infection. Genes were considered differentially expressed if they met the prerequisites *p* ≤ 0.05 and *m* ≤ −0.7 or *m* ≥ 0.7. Up-regulation (*m* ≥ 0.7) is indicated in red; down-regulation (*m* ≤ −0.7) in green; black indicates no differential expression (−0.7 ≤ m ≤ 0.7). The heat map expression profiles are grouped by yellow rectangles (I–X). Additional information is available in Table 1.

We analyzed and compared the expression patterns of TF genes in the susceptible cv. Parabinga and the resistant SA1306 genotypes at 4 h after *E. pisi* inoculation (Figure S1). A total of 623 genes of the qPCR TF platform (59.6%) were considered detected (*Cq* < 40; *n* ≥ 2) and 95 showed statistically significant differences (*P* < 0.05) upon *E. pisi* infection in SA1306 and Parabinga genotypes. The relative gene expression ratios (m), log_2_expression ratios inoculated/control after *E. pisi* inoculation, were calculated for all TF genes. TF genes were considered to be differentially up- or down-regulated in response to *E. pisi* infection, if they met the prerequisites *p* ≤ 0.05 and *m* ≥ 0.7 or *m* ≤ −0.7, respectively. We studied the expression pattern of these genes that showed statistically significant differences through a hierarchical clustering analysis based on gene expression profiles (Figure 2, Table 1).

**Table 1:** **Details of regulated TF genes clustered in groups among** ***Medicago truncatula*** **genotypes analyzed, SA1306 (SA), and Parabinga (PB), in response to** ***Erysiphe pisi*** **infection**.

**ID^a^**	**TC^b^**	**TF family; subfamily^c^**	**Relative gene expression ratios (m)^d^**		**F^e^**	**Group^f^**
			**PB**	**SA**		
TF700	CX528154	BTB/POZ	−2.31^*^	−3.55^*^	−1.24	I
TF479	TC110178	NAC	−1.54^*^	−1.12^*^	0.42	I
TF823	–	C2H2 (Zn)	−1.46^*^	−3.36^*^	−1.89	I
TF436	TC98230	SBP	−1.16^*^	−1.24^*^	−0.08	I
TF855	–	AP2/EREBP	−0.87^*^	−1.49^*^	−0.62	I
TF303	–	AP2/EREBP	−0.86^*^	−1.83^*^	−0.97	I
TF485	TC109384	bHLH	−0.83^*^	−3.51^*^	−2.68	I
TF425	–	bHLH	−0.36	−1.98^*^	−1.62	I
TF101	TC103429	C2H2 (Zn)	−0.28	−1.62^*^	−1.34	I
TF8	TC109476	LIM	−0.28	−1.75^*^	−1.47	I
TF388	BG448434	TTF-type (Zn)	0.61	−2.13^*^	−2.74	I
TF244	TC108091	MYB/HD-like	0.54	−1.04^*^	−1.58	I
TF780	–	AUX/IAA	0.49	−1.21^*^	−1.70	I
TF691	BG450549	E2F	0.49	−1.00^*^	−1.49	I
TF233	–	HMG	0.72^*^	−1.43^*^	−2.15	I
TF962	–	AP2/EREBP	−5.08^*^	1.56^*^	6.64	II
TF626	–	HD-like	−3.62^*^	0.91^*^	4.52	II
TF913	TC97332	WRKY family; WRKY	−1.48^*^	0.77^*^	2.25	II
TF393	TC95605	C2C2 (Zn); DOF	−2.65^*^	−2.19^*^	0.45	II
TF265	–	C2C2 (Zn); DOF	−4.31^*^	−1.42^*^	2.89	II
TF716	AL382911	C2C2 (Zn); GATA	−2.46^*^	−1.60^*^	0.86	II
TF767	–	C3H; C3H-type 1(Zn)	−2.42^*^	0.44	2.86	II
TF822	–	DDT	−1.24^*^	0.69	1.92	II
TF448	TC97611	RR	−1.23^*^	0.06	1.29	II
TF546	AL366881	AP2/EREBP	−0.71^*^	0.26	0.97	II
TF618	–	HD family; HD	−1.03^*^	−0.27	0.76	II
TF598	TC95256	NAC	−0.92^*^	−0.47	0.45	II
TF631	TC96049	HSF	−0.69	−0.47	0.22	II
TF837	–	MYB	5.96^*^	1.87^*^	−4.09	III
TF996	–	HD-like	4.24^*^	3.23^*^	−1.01	III
TF129	TC102127	MYB/HD-like	4.45^*^	2.37^*^	−2.08	III
TF549	TC107542	HD family; HD-ZIP	3.89^*^	3.02^*^	−0.87	III
TF3	–	AP2/EREBP	3.11^*^	1.02^*^	−2.08	III
TF934	TC109302	MYB/HD-like	4.22^*^	0.39	−3.82	III
TF428	–	C2H2 (Zn)	2.88^*^	2.17^*^	−0.71	IV
TF879	CB066652	PHD	2.42^*^	3.53^*^	1.11	IV
TF333	–	bHLH	2.18^*^	1.99^*^	−0.19	IV
TF322	–	MADS	1.98^*^	1.70^*^	−0.28	IV
TF63	–	MYB	1.76^*^	2.80^*^	1.04	IV
TF270	–	C2H2 (Zn)	1.59^*^	4.49^*^	2.90	IV
TF258	–	HD family; HD	1.41^*^	5.26^*^	3.85	IV
TF563	–	MADS	1.25^*^	3.13^*^	1.88	IV
TF230	TC109855	HD-like	0.71^*^	5.66^*^	4.95	IV
TF600	TC103296	bHLH	1.44^*^	1.72^*^	0.29	V
TF87	TC96308	ZF DHHC	1.20^*^	1.40^*^	0.20	V
TF639	TC100932	bHLH	1.32^*^	1.40^*^	0.08	V
TF296	–	GRAS	1.68^*^	1.66^*^	−0.02	V
TF565	–	HTH; FIS	1.57^*^	1.09^*^	−0.48	V
TF386	TC110943	ARF	1.55^*^	0.79^*^	−0.75	V
TF473	TC107897	AP2/EREBP	2.15^*^	1.22^*^	−0.93	V
TF136	TC96243	NAC	2.17^*^	1.21^*^	−0.96	V
TF497	BF636434	HD family; HD	2.08^*^	1.01^*^	−1.07	V
TF276	BE249457	RR	1.71^*^	0.65	−1.06	V
TF364	TC96319	CCAAT; CCAAT-HAP3	−0.20	−0.15	0.05	VI
TF196	TC106782	EIL	−0.05	−0.12	−0.07	VI
TF351	TC103599	bHLH	−0.21	−0.42	−0.21	VI
TF543	TC101251	BD	0.25	−0.44	−0.70	VI
TF449	−	CCHC (Zn)	0.21	−0.59	−0.80	VI
TF438	−	PHD	0.38	−0.43	−0.81	VI
TF899	−	TCP	0.39	−0.51	−0.90	VI
TF441	−	JUMONJI	0.41	−0.54	−0.95	VI
TF959	−	C2H2 (Zn)	0.60	−0.67	−1.28	VI
TF537	−	bZIP	1.09^*^	0.80^*^	−0.29	VII
TF429	TC111833	MYB/HD-like	0.99^*^	0.59	−0.40	VII
TF140	TC107912	PHD	0.86^*^	1.22^*^	0.37	VII
TF97	CX534602	HD-like	0.77^*^	1.28^*^	0.51	VII
TF588	TC106806	HD family; HD	0.73^*^	0.63	−0.10	VII
TF523	TC102139	MYB/HD-like	0.63	0.67	0.04	VII
TF199	−	NAC	0.58	0.55	−0.03	VII
TF464	TC109097	bZIP	0.48	1.05^*^	0.57	VII
TF437	−	CCHC (Zn)	0.47	0.44	−0.03	VII
TF816	TC96871	MYB/HD-like	0.25	0.29	0.04	VII
TF372	TC96859	HD family; HD	0.15	0.97^*^	0.82	VII
TF389	TC104194	C2C2 (Zn); DOF	1.59^*^	−0.06	−1.66	VIII
TF1007	−	bHLH	1.11^*^	0.32	−0.79	VIII
TF349	TC98775	CCHC (Zn)	1.00^*^	0.30	−0.70	VIII
TF179	CX533076	U1-type (Zn)	0.99^*^	0.32	−0.67	VIII
TF481	TC112164	FHA	0.88^*^	−0.03	−0.91	VIII
TF143	−	MYB/HD-like	0.80^*^	0.21	−0.59	VIII
TF797	−	HD-like	1.26^*^	2.47^*^	1.21	IX
TF200	TC109833	NAC	1.10^*^	2.23^*^	1.13	IX
TF814	BG451025	HD-like	1.11^*^	2.45^*^	1.34	IX
TF552	TC107542	HD family; HD-ZIP	0.87^*^	2.09^*^	1.22	IX
TF666	CX541503	HD-like	0.65	2.13^*^	1.48	IX
TF982	TC96831	C2H2 (Zn)	0.52	2.77^*^	2.25	IX
TF901	−	SBP	0.48	2.71^*^	2.23	IX
TF27	−	ARID	0.05	3.00^*^	2.95	IX
TF1009	−	JUMONJI	0.08	1.73^*^	1.65	X
TF726	AL375449	MYB/HD-like	0.45	1.52^*^	1.07	X
TF158	−	HD-like	0.44	1.43^*^	0.99	X
TF540	−	SBP	−0.38	1.31^*^	1.69	X
TF660	−	CCHC (Zn)	−0.83^*^	1.04^*^	1.88	X
TF308	−	C2C2 (Zn); DOF	−0.33	0.75^*^	1.08	X
TF295	−	HD family; HD	−0.35	0.65	1.00	X
TF562	TC98196	HD family; HD	−0.08	0.48	0.55	X

**Figure 3:**
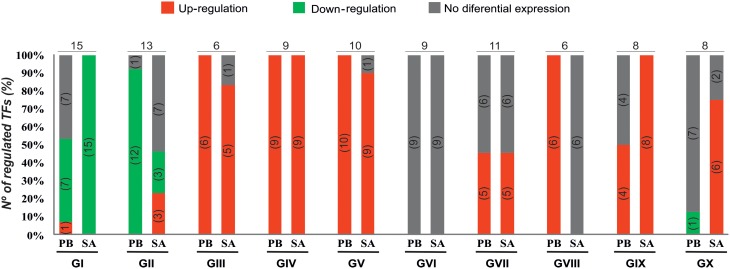
**Expression profiles of TF genes**. Percentages and numbers (into the brackets) of up-regulated (in red), down-regulated (in green), and no differential expressed (in gray) genes detected in Parabinga (PB) and SA1306 (SA) for each of the 10 clusters (groups GI–GX). Top on each cluster the total number of TF regulated genes is indicated. Additional information is available in Table 1.

*Relative gene expression ratios (m) are listed and sorted by cluster (Group)*.

a *TF gene identification number. Additional information is given in Table S1*.

b *Identifier in the TIGR M. truncatula Gene index (MtGI 7)*.

c *TF families; sub-families are showed as described (Kakar et al., 2008)*.

d *Relative gene expression ratios values (m) were calculated for Parabinga (PB) and SA1306 (SA) genotypes, using the following equation: log_2_ differential expression ratio ($\frac{\text{inoculated}}{\text{control}}$)*.

e *Fold change expression ratio (F) in SA1306 compared to Parabinga were calculated using the equation: log_2_ expression ratio ($\frac{\text{SA1306}}{\text{Parabinga}}$)*.

f *Heat map expression profiles clustered. Additional information is given in Figures 2, 3*.

* *Genes were considered differentially expressed in response to E. pisi infection if to meet the prerequisites p ≤ 0.05 and m ≤ −0.7 or m ≥ 0.7*.

### Transcription factor regulatory network induced by *E. pisi* infection in *M. truncatula*

The qPCR platform allowed identification of TF genes differentially expressed in the two *M. truncatula* genotypes in response to *E. pisi* infection. Genes regulated in response to *E. pisi* infection (*p* ≤ 0.05 and *m* ≥ 0.7 or *m* ≤ −0.7) were clustered into 10 groups with different expression patterns (Figure 3). The first group (GI) includes 15 genes that were down-regulated in SA1306. In Parabinga eight of the genes were differentially expressed, one was up-regulated and seven TFs were repressed. These genes belong to 12 TF families, including Zn-Finger members (C_2_H_2_ (CYS_2_-HIS_2_), TTF-type (THYROID TRANSCRIPTION FACTOR), LIM (*L*in-11, *I*sl-1, *M*ec-3), AP2/EREBP (APETALA2/ETHYLENE-RESPONSIVE ELEMENT BINDING FACTORS), AUX/IAA (AUXIN/INDOLE ACETIC ACID), bHLH (BASIC-HELIX-LOOP-HELIX), BTB/POZ (BROAD COMPLEX, TRAMTRACK, BRIC-A-BRAC/POX VIRUS, AND ZINC FINGER), E2F (E2 FACTOR), HMG (HIGH-MOBILITY GROUP), MYB/HD-like (MYELOBLASTOSIS/ HOMEODOMAIN-LIKE), NAC (NAM/ATAF/CUC), and SBP (SQUAMOSA-PROMOTER BINDING PROTEIN) (Table 1). The second group (GII) includes 13 genes which were down-regulated in Parabinga, except for one gene that was not differentially expressed. In the resistant SA1306 genotype, three were up-regulated and other three repressed belong to Zn-finger families, such as C_2_C_2_ (CYS_2_-CYS_2_) and C_3_H-type I (CYS_3_-HIS_1_) family. The remaining genes belong to AP2/EREBP, DDT (DNA BINDING HOMEOBOX, AND DIFFERENT TRANSCRIPTION FACTOR), HD, HD-like, HSF (HEAT STRESS TRANSCRIPTION FACTOR), NAC, RR (RESPONSE REGULATOR RECEIVER), and WRKY TF families.

Groups III, IV, and V include genes that were induced in both genotypes. Group III contains six genes that showed lower transcription levels in SA1306 than in Parabinga. These genes belong to AP2/EREBP, HD, HD-like, MYB, and MYB/HD-like TF families. Group IV includes nine genes that showed stronger transcription activation in the resistant SA1306 genotype. These genes belong to bHLH, C_2_H_2_ (Zn), HD, HD-like, MADS (MADS box), MYB, and PHD (PLANT HOMEODOMAIN MOTIF) TF families. Group V contains 10 genes that showed similar up-regulation expression patterns in both susceptible and resistant genotypes. Genes of this fifth group encode proteins belonging to AP2/EREBP, ARF (AUXIN-RESPONSE FACTOR), bHLH, GRAS (GAI, RGA, SCR), HD, HTH (HELIX-TURN-HELIX), NAC, RR, and DHHC (ASP-HIS-HIS-CYS) (Zn) TF families.

Genes clustered in groups VI and VII were mainly not expressed differentially in either genotype. None of the genes included in group VI were regulated whereas seven of the 11 genes of group VII were regulated. Three genes were induced in both genotypes and they are members of HD-like, PHD, and bZIP (BASIC LEUCINE ZIPPER) TF families, respectively. The remaining four regulated genes of group VII were specifically up-regulated in Parabinga and SA1306, which were included in HD, bZIP, and MYB/HD-like TF families.

Group VIII is comprised of six genes which were induced in Parabinga and not differentially expressed in SA1306. These genes belong to bHLH, FHA (FORKHEAD-ASSOCIATED), MYB/HD-like and three Zn-finger TF families (C_2_C_2_, CCHC (CYS-X8-CYS-X5-CYS-X3-HIS) and U1-type). The eight genes included in group IX were induced in SA1306, whereas in Parabinga half of them were up-regulated and the remaining genes were not differentially expressed. Genes of group IX are members of ARID (AT-RICH INTERACTION DOMAIN), C_2_H_2_ (Zn), HD, HD-like, NAC, and SBP TF families. Finally, group X includes eight genes which were mostly up-regulated in SA1306 and not differentially expressed in Parabinga. All genes belonging to this group were induced in the resistant genotype, except for two genes that were not differentially expressed. Only one repressed gene was detected in Parabinga. The genes of this last cluster belong to Zn finger families C_2_C_2_, CCHC, HD, HD-like, JUMONJI (JmjC domain), MYB/HD-like, and SBP TF families.

**Figure 4:**
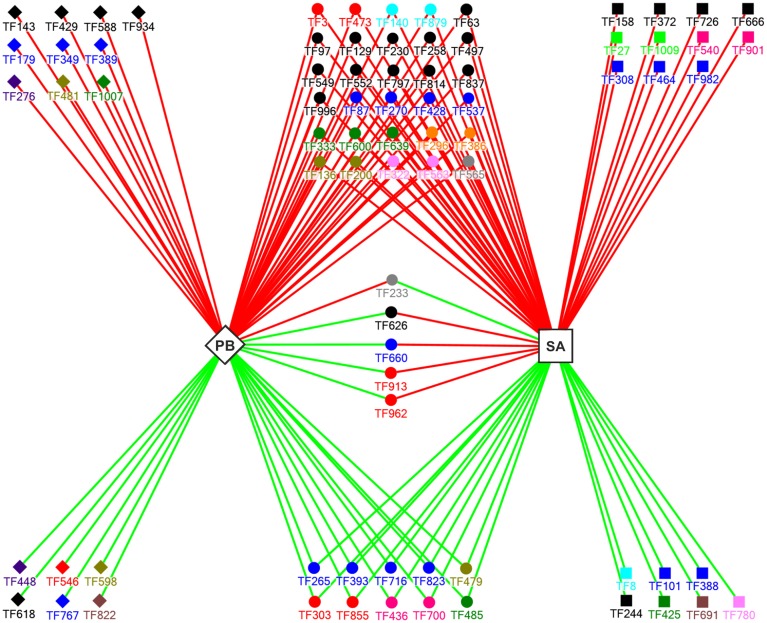
**A model for the regulatory network that controls the expression of** ***E. Pisi*****-induced TF genes (*****p*** **= 0.05; −0.7 ≥ m ≥ 0.7) in Parabinga (PB; diamond) and SA1306 (SA; square)** ***M. truncatula*** **genotypes**. Solid diamonds and solid squares indicate the TF genes regulated in Parabinga and SA1306, respectively. The TF genes that were regulated in both genotypes are indicated by solid spheres. Up- and down-regulation are indicated by red and green lines, respectively. The colors of the solid diamonds, solid squares, and solid spheres indicate TF families: Green (bHLH); black (HD family; HD-Like; MYB; MYB/HD-like); orange (ARF; GRAS); red (AP2/ERBP; WRKY); blue (Zn-fingers TF families; bZIP); olive green (FHA; NAC); violet (RR); pink (SBP; BTB/POZ); gray (HMG; HTH); brown (E2F; DDT); sky-blue (LIM; PHD); pea green (JUMONJI; ARID); dark pink (AUX/IAA; MADS). A detailed description of these genes is shown in Table S2.

Around 80% of TF genes (79/95 genes) that showed statistically significant differences (*P* < 0.05) had at least a 1.6-fold change in transcript accumulation (−0.7 ≥ m ≥ 0.7) (Table S2). To study the regulatory network controlling the expression and interactions of these 79 genes during *E. pisi* infection, we further analyzed their expression in the susceptible Parabinga and the resistant SA1306 genotypes (Figure 4).

Our analysis revealed that 16 and 18 of the 79 TF genes were specifically regulated in Parabinga and in SA1306, respectively. The remaining 45 genes were regulated in both genotypes (Figure 4, Table S2). In the susceptible Parabinga genotype 10 of the 16 specifically regulated genes were induced and the remaining six genes were repressed. Most of the genes specifically induced in Parabinga are members of MYB/HD-like, HD, C_2_C_2_, CCHC, RR, FHA, bHLH, and U1-type Zn finger families. Meanwhile, the six genes specifically down-regulated in Parabinga are included in RR, AP2/EREBP, NAC, HD-family, C_3_H- type 1 (Zn), and DDT TF families. Moreover, the resistant SA1306 genotype showed 11 and seven genes specifically up- and down-regulated, respectively. Genes specifically induced in SA1306 are members of HD/HD-like, MYB/HD-like, SBP, C_2_C_2_ (Zn), C_2_H_2_ (Zn), bZIP, ARID, and JUMONJI TF families. The genes down-regulated in SA1306 belong to LIM, C_2_H_2_ (Zn), TTF-type (Zn), MYB/HD-like, bHLH, E2F, and AUX/IAA TF families.

On the other hand, the genes regulated in both genotypes were mainly up-regulated; 30 induced and 10 repressed genes. Most of these 30 induced genes are in the HD-like, HD, and MYB TF families (Figure 4, Table S2). Moreover, the 10 repressed genes are included mainly in Zn finger families, C_2_C_2_ and C_2_H_2_. Interestingly, four of five common regulated genes were induced in SA1306 and repressed in Parabinga, and belong to the HD-like (TF626), CCHC (Zn) (TF660), WRKY (TF913), and AP2/EREBP (TF962) TF families. The fifth gene, TF233 (HMG), was up-regulated in Parabinga and repressed in SA1306.

**Figure 5:**
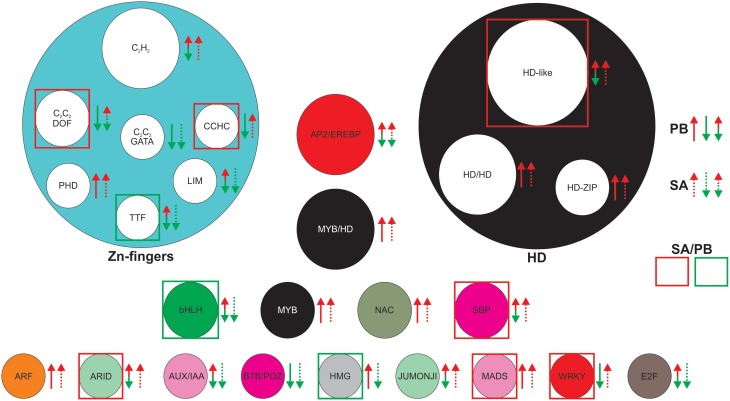
**Most differentially expressed TF families in SA1306 compared to Parabinga (−0.7 >** ***F*** **> 0.7) in response to** ***Erysiphe pisi*** **infection**. The TF families are represented by circles whose size is proportional to the number of genes in the family. Up-regulation (*m* ≥ 0.7) is indicated by red arrows; down-regulation (*m* ≤ −0.7) by green arrows; no differential expression (−0.7 ≤ m ≤ 0.7) are indicate by double red/green arrows; solid lines represent Parabinga (PB), and dotted lines SA1306 (SA). TF families highly up-regulated (*F* ≥ 1.8) and down-regulated (*F* ≤ −1.8) are indicated by red and green boxes, respectively. For expanded information, see Table S3.

Interestingly, 48 genes were differentially expressed in SA1306 compared to Parabinga (*P* < 0.05; −0.7 > *F* > 0.7) in response to *E. pisi* infection, and they belong to 25 TF families (Figure 5, Table S3). Among them, the most represented TF families are HD-like, C_2_H_2_ (Zn), AP2/EREBP, MYB/HD-like, and HD/HD, which comprise approximately half of the differentially expressed genes. The bHLH, C_2_C_2_/DOF (CYS_2_-CYS_2_/DNA-BINDING ONE ZINC FINGER) (Zn), HD-ZIP (HD-ZINC-REGULATED TRANSPORTER), MYB, NAC, and SBP TF families were also well represented (≈24%) and the rest of TF families were poorly represented (Figure 5). Eleven of 25 families include genes with greater transcript accumulation (*F* > 0.7) in SA1306 compared to Parabinga (Figure 5). Among them, the genes belonging to the ARID, WRKY family/WRKY, C_2_C_2_ (Zn)/DOF, SBP, HD-like, MADS, and CCHC (Zn) families were up-regulated around two fold in SA1306 compared to Parabinga. Meanwhile only three families, TTF-type (Zn), bHLH, and HMG, include genes that were down-regulated (*F* ≤ −2) in SA1306 compared to Parabinga (Figure 5). Generally, families HD-ZIP and NAC include genes that were induced in both genotypes. However, the AP2/ERBP family was lightly up-regulated in SA1306 and almost not regulated in Parabinga (Table S3).

## Discussion

Thanks to the high-throughput methods genes, potentially associated with specific processes and characterization of the regulatory networks that control their expression, have been identified (Czechowski et al., 2004; Caldana et al., 2007). DNA microarrays have been successfully applied to characterize global gene expression patterns in *M. truncatula* (Foster-Hartnett et al., 2007; Samac et al., 2011; Zhang et al., 2014; Curto et al., 2015; Song et al., 2015). Previous DNA microarray studies have analyzed the *E. pisi/M. truncatula* pathosystem (Foster-Hartnett et al., 2007; Samac et al., 2011; Curto et al., 2015) using different genotypes and microarray platforms, such as Mt16kOLI1, Mt16kOLI1plus, and Affymetrix GeneChip® (http://www.affymetrix.com), which have increased the knowledge of mechanisms involved in *E. pisi* resistance in *M. truncatula*. Several large-scale TF profiling approaches have employed the *M. truncatula* qPCR-based platform available (Kakar et al., 2008) in various studies (Verdier et al., 2008; Gao et al., 2010; Madrid et al., 2010; Villegas-Fernández et al., 2014; Noguero et al., 2015). In spite of the progress in characterizing TFs, those involved in the expression of stress-related genes in plants remain undiscovered (Singh et al., 2002). Particularly, the TFs involved in the defense mechanisms against *E. pisi* need to be clarified in order to completely understand the mechanisms involved in the plant's defense against this pathogen.

In our study we found that 95 of the TF genes analyzed (15%) were expressed differentially. These results agree with similar approaches carried out in response to infection by *Uromyces striatus* (≈13%) (Madrid et al., 2010) and *Botrytis* spp. (≈20%) (Villegas-Fernández et al., 2014). A subset of these genes belong to 25 TF families (Figure 5, Table S3), including AUX/IAA, bHLH, E2F, HD, JUMONJI, MYB, SBP, and zinc finger families (C_2_C_2_, C_2_H_2_, LIM), were specifically regulated in the resistant SA1306 genotype suggesting that they act as major regulators of transcription throughout *E. pisi* defense responses.

Zinc finger and HD families represent the most of these genes specifically regulated in the resistant SA1306 genotype, and are members of the C_2_H_2_ (Zn), C_2_C_2_/DOF, LIM, HD-like, HD/HD, and HD-ZIP TF families, which are agree with recent studies that used the same qPCR TF platform (Villegas-Fernández et al., 2014). The C_2_H_2_ (Zn) family playing a critical role as key transcriptional repressors involved in the defense response of plants to stress (Brayer and Segal, 2008; Ciftci-Yilmaz and Mittler, 2008; Kiełbowicz-Matuk, 2012) and *M. truncatula* to biotrophic and necrotrophic pathogens. Nevertheless, previous studies have reported the relationship between SBP genes and plant disease resistance, such as programmed cell death in *Arabidopsis* (Stone et al., 2005) and these studies are in agreement that programmed cell death, frequently associated to host cell death, is a common plant defense mechanisms against *E. pisi* (Curto et al., 2006; Barilli et al., 2014). Thus, a member of the SPB TF family (*VpSBP5*) has been reported to be induced by powdery mildew (*E. necator*) (Hou et al., 2013), suggesting that this TF is involved in the resistance to powdery mildew by inducing salicylic acid and methyl jasmonate molecular signals. The C_2_C_2_ (Zn)/DOF family member have divergent physiological roles (Yanagisawa, 2002) including defense gene expression in response to salicylic acid and oxidative stress signals (Chen et al., 1996; Yanagisawa, 2002) and phytohormone-regulated expression (De Paolis et al., 1996; Yanagisawa, 2002). Recent studies have shown that *E. pisi* infection induces several enzymes, such as *psCHS1* and *PEAPAL2*, involved in phenylpropanoid biosynthesis leading to biosynthesis of phytoalexin (Barilli et al., 2014), reinforcing the role of phenylpropanoid pathway in the elicited defense. Our findings are in agreement with these previous studies, suggesting the important role of these TF families in the resistance to *E. pisi* in *M. truncatula*.

Moreover, the LIM zinc TF family has also been found to be involved in the resistance mechanism against pathogens. Thus, several studies have revealed that this family has the capacity to regulate the expression of some lignin biosynthetic genes (Rogers and Campbell, 2004). In addition, previous studies have reported the role of lignification processes into the *M. truncatula* defense responses against to *E. pisi* infection (Prats et al., 2007). Our findings are in agreement with these previous studies and recent studies (Barilli et al., 2014), in which enzymes involved in ROIs stress, such as peroxidase *Prx7*, are regulated in pea after *E. pisi* inoculation. Other studies have found that monolignol biosynthesis plays a critical role in cell wall apposition mediated defense against powdery mildew fungus penetration (Bhuiyan et al., 2009). Reactive oxygen intremediates (ROIs) are associated with the hypersensitive response (Torres et al., 2005), which has been related to programmed cell death (hypersensitive response) that plays a critical role in resistance to *E. pisi* (Barilli et al., 2014).

Our results also reveal a subset of TF genes that encode TFs with a HD (homeodomain) protein domain, which showed a different expression pattern in the resistant SA1306 compared to the susceptible Parabinga genotype in response to *E. pisi* infection. These genes belong to the HD-like and HD family/HD. Members of this family may play a role in the defense response against necrotrophic fungal pathogens regulated by jasmonic acid (Korfhage et al., 1994; Coego et al., 2005; Villegas-Fernández et al., 2014). In addition, a recent study has revealed that several homeodomain-like TF families are involved in the defense responses in *M. truncatula* when confronted with the necrotrophic pathogens, *Botrytis fabae*, and *B. cinerea* (Villegas-Fernández et al., 2014), supporting that these TF families may play an important role in the defensive mechanism of *M. truncatula* to fungal pathogens. Nevertheless, our results show that the Auxin/Indole-3-acetic acid (AUX/IAA) family was repressed in the resistant SA1306 genotype in response to *E. pisi* infection, which suggests that this family may play a role in the resistance mechanism against *E. pisi*. Earlier studies are agree with our results and have described that down regulation of auxin signaling contribute to plant induced immune responses in *Arabidopsis* (Navarro et al., 2006). The bHLH TFs were also induced in the resistant genotype in response to *E. pisi* infection, which are in agreement with the results obtained by Villegas-Fernández et al. (2014). The bHLH TFs up-regulated by *E. pisi* key TF regulating the expressions of jasmonic acid responsive genes (Fernández-Calvo et al., 2011), which mediate the transcriptional reprogramming associated with the plant immune response.

Our findings also indicate that E2F and JUMONJI TF families are involved in the defense response against *E. pisi*. The E2F transcription factor family has been found to induce transcription of genes required for cell cycle progression and DNA replication (Vandepoele et al., 2005). In addition, more than 100 E2F target genes have been identified, including genes involved in several defense responses and signaling (Ramirez-Parra et al., 2003). The members if the family JUMONJI play a role in the histone methylation process (Li et al., 2013). Dimethylated or trimethylated histone H3 lysine 27 (H3K27me2/3) marks silent or repressed genes involved in stress responses in plants. Li et al. (2013) studied the *jumonji C* protein gene *JMJ705* that is induced by stress signals during pathogen infection, and is involved in methyl jasmonate–induced dynamic removal of H3K27me3 and in gene activation increasing their basal and induced expression during pathogen infection (Balciunas and Ronne, 2000; Li et al., 2013). Our results are in agreement with the results obtained by Villegas-Fernández et al. (2014), which It is that have reported that the JUMONJI TF family may be involved in the defense response to a fungal pathogen, reinforcing the role of this TF family in defense responses against fungal pathogens.

Moreover, an important number of TF genes were regulated in both genotypes, which mainly belonged to AP2/EREBP (Singh et al., 2002; Gutterson and Reuber, 2004; Dietz et al., 2010; Villegas-Fernández et al., 2014), C_2_H_2_ (Zn) (Ciftci-Yilmaz and Mittler, 2008; Villegas-Fernández et al., 2014), MYB (Singh et al., 2002; Villegas-Fernández et al., 2014), HD (Coego et al., 2005; Villegas-Fernández et al., 2014), MYB/HD-like (Singh et al., 2002; Coego et al., 2005), NAC (Dangl and Jones, 2001; Villegas-Fernández et al., 2014), and PHD (Libault et al., 2007; Villegas-Fernández et al., 2014) TF families. Interestingly, the susceptible and resistant genotypes showed different expression patterns in five of these TF genes in response to *E. pisi* infection (Figure 4). Four of these five genes (TF626, TF660, TF913, and TF962) encode TFs belonging to known defense system pathways, which were induced in SA1306 and repressed in Parabinga, respectively. TF962 encodes a member of the AP2/EREBP family known to be linked to response to abiotic and biotic stresses (Dietz et al., 2010), as well as involved in response to a chitin elicitor and in metabolism of the plant hormone methyl jasmonate (McGrath et al., 2005; Libault et al., 2007). Results obtained were similar to those generated in of a study of *M. truncatula* of responses to *Botrytis* infection, in which AP2/EREBP was shown to be a key regulator of defense responses (Villegas-Fernández et al., 2014). TF913 encodes a WRKY protein, whose family members have been shown to be key components in the regulation of plant disease resistance (Eulgem and Somssich, 2007). Previous studies have shown that WRKY TF family members are involved in the regulation of *R* gene-mediated disease resistance as well as in the regulation of transcriptional reprogramming associated with plant immune responses (Eulgem and Somssich, 2007; Buscaill and Rivas, 2014). Several genes encoding WRKY proteins (*AtWRKY18, AtWRKY40*) have been identified that confer resistance against powdery mildew (Shen et al., 2007). In addition, *AtWRKY18* has been characterized to act as a positive regulator required for full SAR (Wang et al., 2006), whose transcriptional expression may be is linked to *AtWRKY70*, which modulates the cross-talk between signaling pathways regulating salicylic acid (SA)-dependent and jasmonic acid-dependent responses (Eulgem and Somssich, 2007). These results are in accord with results of recent studies in *M. truncatula* that WRKY TFs involved in the defensive reaction of *M. truncatula* to *Uromyces* and *Botrytis* (Madrid et al., 2010; Villegas-Fernández et al., 2014), supporting the critical role of this TF family in the plant defense responses against fungal pathogens.

The Zn finger family CCHC (TF660) was also detected as induced in SA1306 and repressed in Parabinga. Previous studies (Mangeon et al., 2010; Villegas-Fernández et al., 2014) are in agreement with our results, suggesting the important role of this TF family, A member of HD-like (TF626) was also induced in the resistant genotype and highly repressed in Parabinga. This family has been found to be involved in defense responses against fungal necrotroph pathogens regulated by jasmonic acid (Korfhage et al., 1994; Coego et al., 2005; Villegas-Fernández et al., 2014). We suggest that the 10 TFs that were repressed in both genotypes may play a role suppressing genes involved in photosynthetic metabolism leading to a reduction in the photosynthetic rate, as previously suggested (Swarbrick et al., 2006; Bolton, 2009).

## Conclusion

We have screened more than 1000 TFs genes of *M. truncatula* for altered expression during *E. pisi* infection using qPCR platform. Forty eight TFs from them showed significant differences in the resistant SA1306 genotype compared to the susceptible Parabinga. These TF genes belong mainly to AP2/EREBP, WRKY, MYB, HD, and zinc finger families (C2C2, C2H2, LIM) gene families, which are involved in known defense responses. In addition, we suggest a regulatory network that controls the expression in *M. truncatula* of genes involved in resistance to *E. pisi*. These results will help to systematically decipher the functional roles of TF genes and to develop new strategies against powdery mildew.

### Conflict of interest statement

The authors declare that the research was conducted in the absence of any commercial or financial relationships that could be construed as a potential conflict of interest.

## Supplementary material

Figure S1 **Macroscopic disease symptoms in the susceptible cultivar Parabinga (A) and in the resistant SA1306 accession (B)**.

Table S1 **Complete list of TF genes**. The accession numbers (AC), the TIGR *Medicago* gene accession numbers (TC) if available, as well as the transcription factor family and the subfamily names are shown as described Kakar et al. (Kakar et al., 2008). The quantification cycle (Cq), PCR efficiencies (PCReff) and correlation coefficients for the qPCR reactions performed on both *Medicago truncatula* genotypes, Parabinga and SA1306, in three biological replicates are indicated.

Table S2 **Details of regulated TF genes among *Medicago truncatula* genotypes analyzed, SA1306 (SA) and Parabinga (PB), in response to *Erysiphe pisi* infection (p ≤ 0.05; −0.7 ≥ m ≥ 0.7)**. Relative gene expression ratios (m) are listed and sorted by “Class” and “m” values. ID: Identification number of TF gene. TF family: Transcription factor family. TF subfamily: Subfamily names. m: Average log_2_ differential expression ratios (inoculated/control) for Parabinga (PB) and SA1306 (SA) genotypes. F: Fold change expression ratios of differentially expressed genes in SA1306 compared to Parabinga, log_2_ expression ratio SA1306/Parabinga. Class: Genes that were specifically regulated in SA1306 (SA), Parabinga (PB) and both genotypes (Common) (Figure 4).

Table S3 **Most differentially expressed TF families in SA1306 compared to Parabinga (−0.7>F>0.7) in response to *Erysiphe pisi* infection**.
