# Supplementary figures and images for: Transcriptional profiling of Medicago truncatula during Erysiphe pisi infection
Source: Front Plant Sci. 2015 Jul 9;6:517. doi: 10.3389/fpls.2015.00517 (PMC4496563; doi:10.3389/fpls.2015.00517)

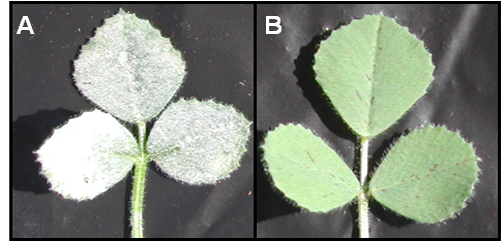

Supplement: Figure S1 — Macroscopic disease symptoms in the susceptible cultivar Parabinga (A) and in the resistant SA1306 accession (B). [file Image1.TIF]
