# Supplementary material for: Transcriptional profiling of Medicago truncatula during Erysiphe pisi infection
Source: Front Plant Sci. 2015 Jul 9;6:517. doi: 10.3389/fpls.2015.00517 (PMC4496563; doi:10.3389/fpls.2015.00517)
Supplement: Table S3 — Most differentially expressed TF families in SA1306 compared to Parabinga (−0.7>F>0.7) in response to Erysiphe pisi infection. [file Table3.DOCX]

Table S3 Most differentially expressed TF families in SA1306 compared to Parabinga (-0.7˃F˃0.7) in response to *Erysiphe pisi* infection.

| **TF family; subfamily ^a^** | **ID ^b^** | **InterPro No ^c^** | **Domain Function ^d^** | **Expression pattern** | | | | | | | |
| --- | --- | --- | --- | --- | --- | --- | --- | --- | --- | --- | --- |
|  |  |  |  | **PB** | |  | **SA** | |  | **SA/PB** | |
|  |  |  |  | **m ^e^** | $\bar{\boldsymbol{x}}$ **^f^** |  | **m ^e^** | $\bar{\boldsymbol{x}}$ **^f^** |  | **F ^g^** | $\bar{\boldsymbol{x}}$ **^h^** |
| **AP2/EREP** | TF3 | **IPR001471** | **D** | 3,11 | -0,17 |  | 1,02 | 0,49 |  | -2,08 | 0,66 |
|  | TF303 |  |  | -0,86 |  |  | -1,83 |  |  | -0,97 |  |
|  | TF473 |  |  | 2,15 |  |  | 1,22 |  |  | -0,93 |  |
|  | TF962 |  |  | -5,08 |  |  | 1,56 |  |  | 6,64 |  |
| **ARF** | TF386 | **IPR003340 IPR010525 IPR011525** | **D** | 1,55 | nc* |  | 0,79 | nc* |  | -0,75 | nc* |
| ARID | TF27 | IPR001606 | D | 0,05 | nc* |  | 3,00 | nc* |  | 2,95** | nc* |
| **AUX/IAA** | TF780 | **IPR003311** | **D** | 0,49 | nc* |  | -1,21 | nc* |  | -1,70 | nc* |
| bHLH | TF425 | IPR001092 | D | -0,36 | -0,60 |  | -1,98 | -2,74 |  | -1,62 | -2,15** |
|  | TF485 |  |  | -0,83 |  |  | -3,51 |  |  | -2,68 |  |
| BTB/POZ | TF700 | IPR000210 | P | -2,31 | nc* |  | -3,55 | nc* |  | -1,24 | nc* |
| ***C_2_C_2_ (Zn); DOF*** | TF265 | ***IPR003851*** | ***D*** | -4,31 | -2,32 |  | -1,42 | -0,34 |  | 2,89 | 1,98** |
|  | TF308 |  |  | -0,33 |  |  | 0,75 |  |  | 1,08 |  |
| *C_2_C_2_ (Zn); GATA* | TF716 | *IPR000679* | *D* | -2,46 | nc* |  | -1,60 | nc* |  | 0,86 | nc* |
| *C_2_H_2_ (Zn)* | TF101 | *IPR007087* | *NA* | -0,28 | 0,65 |  | -1,62 | 0,89 |  | -1,34 | 0,24 |
|  | TF270 |  |  | 1,59 |  |  | 4,49 |  |  | 2,90 |  |
|  | TF428 |  |  | 2,88 |  |  | 2,17 |  |  | -0,71 |  |
|  | TF823 |  |  | -1,46 |  |  | -3,36 |  |  | -1,89 |  |
|  | TF982 |  |  | 0,52 |  |  | 2,77 |  |  | 2,25 |  |
| *CCHC (Zn)* | TF660 | *IPR001878* | *NA* | -0,83 | nc* |  | 1,04 | nc* |  | 1,88** | nc* |
| E2F | TF691 | IPR003316 | D | 0,49 | nc* |  | -1,00 | nc* |  | -1,49 | nc* |
| HD family; HD | TF258 | IPR001356 | D | 1,41 | 1,21 |  | 5,26 | 2,41 |  | 3,85 | 1,20 |
|  | TF372 |  |  | 0,15 |  |  | 0,97 |  |  | 0,82 |  |
|  | TF497 |  |  | 2,08 |  |  | 1,01 |  |  | -1,07 |  |
| HD family; HD-ZIP | TF549 | IPR006712 | P | 3,89 | 2,38 |  | 3,02 | 2,55 |  | -0,87 | 0,17 |
|  | TF552 |  |  | 0,87 |  |  | 2,09 |  |  | 1,22 |  |
| HD-like | TF158 | IPR009057 | D | 0,44 | 0,68 |  | 1,43 | 2,61 |  | 0,99 | 1,93** |
|  | TF230 |  |  | 0,71 |  |  | 5,66 |  |  | 4,95 |  |
|  | TF626 |  |  | -3,62 |  |  | 0,91 |  |  | 4,52 |  |
|  | TF666 |  |  | 0,65 |  |  | 2,13 |  |  | 1,48 |  |
|  | TF797 |  |  | 1,26 |  |  | 2,47 |  |  | 1,21 |  |
|  | TF814 |  |  | 1,11 |  |  | 2,45 |  |  | 1,34 |  |
|  | TF996 |  |  | 4,24 |  |  | 3,23 |  |  | -1,01 |  |
| **HMG** | TF233 | IPR000637 | D | 0,72 | nc* |  | -1,43 | nc* |  | -2,15** | nc* |
| **JUMONJI** | TF1009 | IPR003347 | D | 0,08 | nc* |  | 1,73 | nc* |  | 1,65 | nc* |
| ***LIM*** | TF8 | *IPR001781* | *P* | -0,28 | nc* |  | -1,75 | nc* |  | -1,47 | nc* |
| **MADS** | TF563 | IPR002100 | D | 1,25 | nc* |  | 3,13 | nc* |  | 1,88** | nc* |
| MYB | TF63 | IPR001005 | D | 1,76 | 3,86 |  | 2,80 | 2,34 |  | 1,04 | -1,52 |
|  | TF837 |  |  | 5,96 |  |  | 1,87 |  |  | -4,09 |  |
| MYB/HD-like | TF129 | IPR001005 IPR009057 | D | 4,45 | 1,81 |  | 2,37 | 0,95 |  | -2,08 | -0,86 |
|  | TF244 |  |  | 0,54 |  |  | -1,04 |  |  | -1,58 |  |
|  | TF726 |  |  | 0,45 |  |  | 1,52 |  |  | 1,07 |  |
| **NAC** | TF136 | **IPR003441** | **D** | 2,17 | 1,64 |  | 1,21 | 1,72 |  | -0,96 | 0,09 |
|  | TF200 |  |  | 1,10 |  |  | 2,23 |  |  | 1,13 |  |
| *PHD* | TF879 | *IPR001965* | *P* | 2,42 | nc* |  | 3,53 | nc* |  | 1,11 | nc* |
| **SBP** | TF540 | **IPR004333** | **D** | -0,38 | 0,05 |  | 1,31 | 2,01 |  | 1,69 | 1,96** |
|  | TF901 |  |  | 0,48 |  |  | 2,71 |  |  | 2,23 |  |
| TTF-type (Zn) | TF388 | IPR006580 | D | 0,61 | nc* |  | -2,13 | nc* |  | -2,74** | nc* |
| **WRKY family; WRKY** | TF913 | **IPR003657** | **D** | -1,48 | nc* |  | 0,77 | nc* |  | 2,25** | nc* |

^a^ TF families; sub-families are showed as described ([Kakar et al. 2008](#_ENREF_28)). Plant-specific families are indicated in **bold**. Zn fingers TF families are marked in *cursive*.

^b^ TF gene identification number. Additional information is given in Table S1.

^c^ Accession numbers of InterPro database (<http://www.ebi.ac.uk/interpro/>).

^d^ D = DNA binding domain; P = protein-protein interaction domain; NA = nucleic acid (DNA and RNA) binding domain; RD = receiver domain

^e^ Relative gene expression ratios values (m) were calculated for Parabinga (PB) and SA1306 (SA) genotypes, using the following equation: $\log_{2} differential expression ratio (\frac{\mathrm{inoculated}}{\mathrm{control}})$.

^f^ Mean of m values of genes included in each TF family.

^g^ Fold change expression ratio (F) in SA1306 compared to Parabinga were calculated using the equation: $\log_{2} expression ratio (\frac{SA1306}{\mathrm{Parabinga}})$

^h^ Average F values of genes included in each TF family.

*nc: value non-calculated.

** TF family highly regulated in SA1306 compared to Parabinga (-1.8≥F≥1.8) in response to *E. pisi* infection.
